# Theoretical Modeling of Intensity Noise in InGaN Semiconductor Lasers

**DOI:** 10.1155/2014/475423

**Published:** 2014-07-22

**Authors:** Moustafa Ahmed

**Affiliations:** Department of Physics, Faculty of Science, King Abdulaziz University, P.O. Box 80203, Jeddah 21589, Saudi Arabia

## Abstract

This paper introduces modeling and simulation of the noise properties of the blue-violet InGaN laser diodes. The noise is described in terms of the spectral properties of the relative intensity noise (RIN). We examine the validity of the present noise modeling by comparing the simulated results with the experimental measurements available in literature. We also compare the obtained noise results with those of AlGaAs lasers. Also, we examine the influence of gain suppression on the quantum RIN. In addition, we examine the changes in the RIN level when describing the gain suppression by the case of inhomogeneous spectral broadening. The results show that RIN of the InGaN laser is nearly 9 dB higher than that of the AlGaAs laser.

## 1. Introduction

InGaN laser diodes have been the subject of considerable attention because of their applications in high density optical disc storage and optical data processing. In particular, blue-violet laser diodes operating at a wavelength around 400 nm are required for Blu-ray disc systems if the disk storage capacity is to be increased up to 25 Gbytes [1]. Much progress in the developments of violet-blue lasers has been made since the first operation of such a laser was reported by Nakamura et al. [2, 3]. Meanwhile, several groups have reported continuous-wave operation at room temperature using different fabrications methods [4–8].

A typical limiting factor for the optic-disc application is the noise in the laser emission, which may cause errors in the reading/recording processes. Semiconductor laser radiation intrinsically shows intensity and phase fluctuations which induce broadening of the spectral line. These fluctuations are associated with quantum transitions of charge carriers between the conduction and valence bands through the recombination processes of charge carriers and the processes of photon emission and absorption [9]. This intrinsic noise is unavoidable and is called quantum noise [10] or “optical shot noise” [11]. The laser noise may be amplified by other effects such as competition among the oscillating modes [12], external modulation [13], and external optical feedback [14]. These types of noise have been intensively investigated in both theory and experiment for near infrared GaAs and InP-based laser diodes [14–20]. Experimental studies on the noise of blue-violet InGaN lasers showed that the properties of noise in the blue-violet laser are not so different qualitatively from those in the infrared lasers [11]. However, the quantum noise in the blue-violet laser is eight times higher than that in the near infrared lasers in terms of RIN at the same output power [11]. To the best of the authors' knowledge, no theoretical investigations on the noise issue in these short-wavelength laser candidates have been reported.

The dynamics and noise of semiconductor lasers are described by a set of stochastic rate equations that describe the mechanisms of time evolution of the adding/dropping of photons and charge carriers [21]. The intensity noise is characterized in terms of the spectral properties of RIN. Analysis of noise and dynamics in semiconductor lasers is dependent on the form of the optical gain in the rate equations and more realistic model should include the effect of the gain suppression [22]. Abdullah [23] indicated that the gain suppression has an important effect on the dynamics of InGaN-based laser diodes, because these lasers may operate with high power where the gain suppression is pronounced. Ahmed and Yamada [24] showed that, in the limit of high power, nonlinear gain is inaccurately described by the commonly used third-order gain expression, which corresponds to partial homogeneous broadening of gain [25]. Instead, the nonlinear gain expression should include higher-order terms from the infinite gain expansion in terms of the electric field intensity to account for the case of high power emission [23].

In this paper, we introduce modeling and simulation on the spectral properties of RIN in the blue-violet InGaN laser diode. The studies are based on appropriate modeling of the rate equations and intensive computer simulations of the laser dynamics and noise. We enhance the novelty of the present work by comparing the simulated results with the previous publications such as in [11]. In addition, we compare the noise results of 410 nm InGaN lasers with those of 830 nm AlGaAs lasers. Also, we study the influence of gain suppression on the noise properties and compare the noise results when using the expressions of inhomogeneous gain broadening. We show that RIN is suppressed remarkably with the increase in the injection current in the regime near the threshold level and that the RIN level in the InGaN laser is nearly 9 dB higher than that of the AlGaAs laser. The noise results are compared with the experimental results in [11]. The increase in the gain suppression was found to suppress the quantum noise within 1 dB/Hz. Finally, we point out that the case of inhomogeneously broadened gain overestimates the RIN level by about 4 dB in the limit of high power emission.

## 2. Theoretical Model

The noise properties of the InGaN laser are described by solving the following rate equations of the photon number *S*(*t*) and injected carriers *N*(*t*) in the active region at a given current *I*:

(1) $$\begin{array}{c} \frac{dS}{dt}=\left(G-G_{\text{th}}\right)S+\frac{a\xi }{V}N+F_{S}\left(t\right)\\ \frac{dN}{dt}=\frac{I}{e}-\frac{N}{\tau_{s}}-G_{L}S+F_{N}\left(t\right), \end{array}$$

where *G* is the optical gain and is composed of linear term *G*_*L*_ and nonlinear term *G*_*NL*_ [25],

(2) $$\begin{array}{c} G=G_{L}-G_{NL}S. \end{array}$$

The linear term *G*_*L*_ is a linear function of *N* and is characterized by the gain slope *a* and the injected carrier number at transparency *N*_*g*_ as [25]

(3) $$\begin{array}{c} G_{L}=\frac{a\xi }{V}\left(N-N_{g}\right), \end{array}$$

whereas the nonlinear gain *G*_*NL*_ is given in terms of the injected carrier number *N* as [25]

(4) $$\begin{array}{c} G_{NL}=B_{c}\left(N-N_{s}\right), \end{array}$$

where *B*_*c*_ and *N*_*s*_ are characteristic parameters of the nonlinear gain. The influence of the nonlinear gain is to suppress the optical gain under the threshold gain level *G*_th_ due to the increase in the photon number *S* when the laser operates above threshold [24]. In (3), *ξ* is the confinement factor of the optical field in the active layer whose volume is *V*. The other parameters in (1) include *e* as the electron charge and *τ*_*s*_ as the electron lifetime due to the spontaneous emission. The last terms *F*_*S*_(*t*) and *F*_*S*_(*t*) are Langevin noise sources with zero mean values and are added to the equations to account for intrinsic fluctuations in *S*(*t*) and *N*(*t*), respectively [9]. These noise sources are assumed to have Gaussian probability distributions and to be *δ*-correlated processes [9]. The frequency content of intensity fluctuations is measured in terms of RIN, which is calculated from the fluctuations $\delta S\left(t\right)=S\left(t\right)-\bar{S}$ in *S*(*t*), where $\bar{S}$ is the time-average value of *S*(*t*). Over a finite time *T*, RIN is given as [14]

(5) $$\begin{array}{c} \text{RIN}=\frac{1}{{\bar{S}}^{2}}\left\{\frac{1}{T}{\left|\overset{T}{\underset{0}{\int }}\delta S\left(t\right)e^{-j2\pi f\tau }d\tau \right|}^{2}\right\}, \end{array}$$

where *f* is the noise frequency. The noise performance of the laser is evaluated also in terms of the average value of the RIN components at frequencies lower than 100 MHz, LF-RIN. The power *P*(*t*) emitted from the front facet is determined from the photon number *S*(*t*) as [9]

(6) $$\begin{array}{c} P\left(t\right)=\frac{hc^{2}}{2n_{D}L_{D}\lambda }\frac{\left(1-R_{f}\right)\ln\left(1/R_{f}R_{b}\right)\;}{\left(1-\sqrt{R_{f}R_{b}}\right)\left(1-\sqrt{R_{f}R_{b}}\right)}S\left(t\right), \end{array}$$

where *λ* is the emission wavelength, *h* is Planck's constant, and *R*_*f*_ and *R*_*b*_ are the power reflectivities at the front and the back facets, respectively.

## 3. Numerical Calculations

Rates (1) are solved by the 4th order Runge-Kutta method using a time integration of Δ*T* = 10 ps. At each integration instant, the noise sources *F*_*S*_(*t*) and *F*_*N*_(*t*) are generated following the technique developed in [14] using two uniformly distributed random numbers generated by the computer. RIN of the total output is calculated from the fast Fourier transform (FFT) of the time fluctuations *δS*(*t*_*i*_) via (5) as follows:

(7) $$\begin{array}{c} \text{RIN}=\frac{1}{{\bar{S}}^{2}}\frac{\Delta t^{2}}{T}{\left|\text{FFT}\left[\delta S\left(t_{i}\right)\right]\right|}^{2}. \end{array}$$

In the calculations, we assume 410 nm multiple quantum well (MQW) InGaN single mode laser with the parameters listed in Table 1. The active region is assumed to contain three quantum wells (QWs) with well thickness, barrier thickness, and stripe width of 5 nm, 10 nm, and 5 *μ*m, respectively. The optical confinement factor in each quantum well layer is 0.0342.

## 4. Results and Discussion

### 4.1. RIN in InGaN Laser Diodes

**Figure 1:**
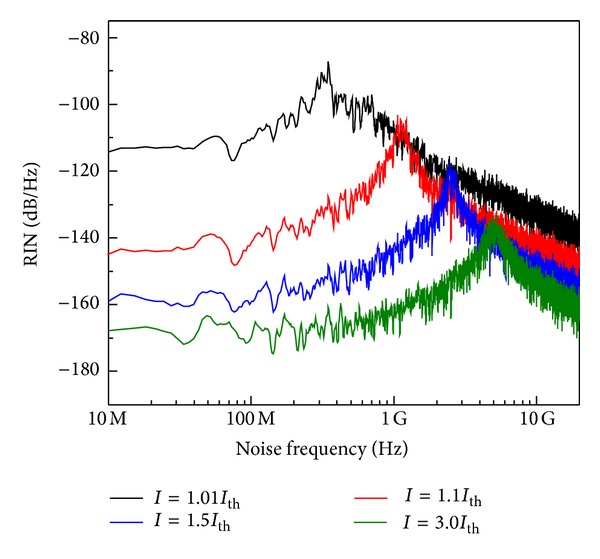
Spectra of RIN as functions of *I*/*I*_th_ for InGaN lasers.

Figure 1 plots the spectral characteristics of RIN at different injection levels: near above threshold (*I* = 1.01 and 1.1*I*_th_), above threshold (*I* = 1.5*I*_th_), and far above threshold (*I* = 3.0*I*_th_). The RIN spectra are flat (white noise) in the low-frequency regime due to the small amplitude of the intensity fluctuations and the large signal-to-noise ratio. In the high-frequency regime, the spectra exhibit the well-known carrier-photon resonance peak around the relaxation frequency *f*_*r*_. The figure shows suppression of RIN with the increase in the injection current *I* due to the improvement in the degree of laser coherency [9]. This increase in the injection level is associated also with an increase in the relaxation oscillation peak; *f*_*r*_ = 330 MHz when *I* = 1.01*I*_th_ and *f*_*r*_ = 5.1 GHz when *I* = 3.01*I*_th_.

**Figure 2:**
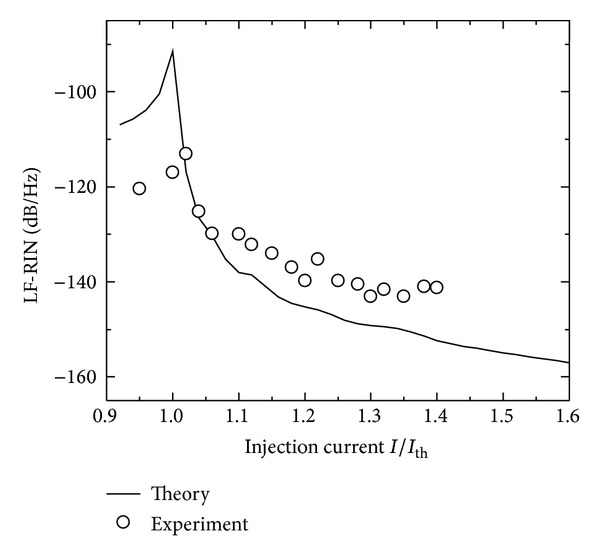
Variation of LF-RIN with the injection current *I*/*I*_th_ of InGaN lasers (solid). The experimental results in [11] are shown (circles) for comparison.

It is of practical interest to study variation of the low frequency level of RIN and LF-RIN with the increase in the injection current *I*.  Figure 2 plots such a variation, showing that LF-RIN increases with the increase in *I* around the threshold current *I*_th_. The further increase in *I* results in a drop of LF-RIN to lower orders of magnitude up to *I* ~ 1.6*I*_th_ (LF-RIN decreases from the peak of −91.8 dB/Hz when *I* = *I*_th_ to −157 dB/Hz when *I* = 1.6*I*_th_). When *I* increases beyond 1.6*I*_th_, the decrease in LF-RIN with the increase in *I* becomes as small as ~0.5 dB/Hz. This suppression of RIN is associated with a decrease in the amplitude of intensity fluctuations in the laser signal and improvement in the signal to noise ratio [9]. We plot also in Figure 2 the experimental results on the quantum noise of InGaN laser reported by Matsuoka et al. [11]. The figure shows that the simulated noise results fit qualitatively the experimental data in [11]. The differences in the noise level between the simulated and experimental results could be a mode competition effect in the measured laser, which may change the noise level of the modeled single mode laser [26].

### 4.2. Comparison of RIN in InGaN Lasers with InGaN and AlGaAs Lasers

**Figure 3:**
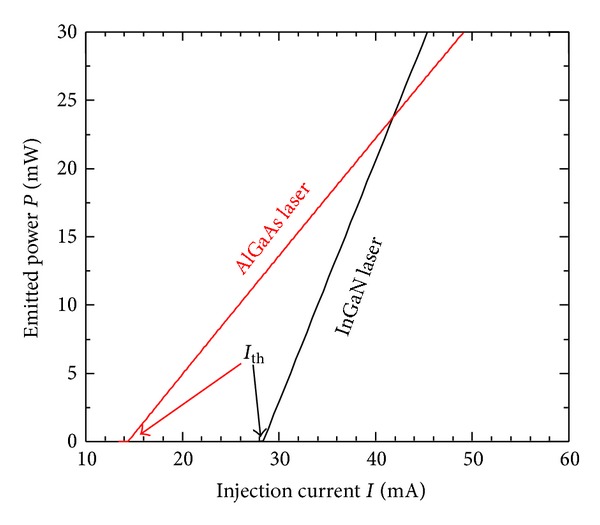
The *L*-*I* characteristics of the InGaN and AlGaAs laser diodes.

In this subsection, we compare the lasing characteristics of the 410 nm GaInN laser with those of 830 nm AlGaAs laser diode. For AlGaAs laser, we consider the following parametric values: *a* = 2.7 × 10^−12^ m^2^, *n*_*D*_ = 3.59, *N*_*g*_ = 1.89 × 10^8^, *N*_*s*_ = 1.63 × 10^8^, *B*_*c*_ = 3.95 × 10^−5^ s^−1^, and *τ*_*s*_ = 2.7 ns.  Figure 3 compares the *L*-*I* characteristics of the two laser diodes showing that the laser operation is linear over the relevant range of injection current *I*. The threshold current *I*_th_, which is the intercept of the *L*-*I* line with the current axis, is shown to increase from 14.2 mA in the AlGaAs laser to 28.2 mA in the InGaN laser.

**Figure 4:**
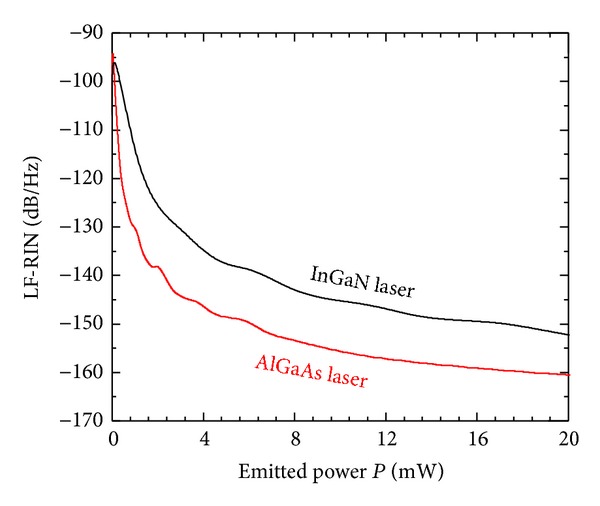
Variation of LF-RIN as a function of the emitted power *P*.

Figure 4 plots variation of the LF-RIN level with the emitted power for the two laser diodes. The figure shows the effect of noise suppression with the increase in the lasing power *P*, with the suppression being stronger in the regime of the near above threshold. The figure shows that LF-RIN in the InGaN laser is nearly 9 dB higher than that of the AlGaAs laser. This higher level of the LF-RIN in the InGaN laser is due to the inverse proportionality of RIN on the third power of the emission wavelength *λ* [10]:

(8) $$\begin{array}{c} \text{RIN}\propto \frac{1}{\lambda^{3}P^{3}}. \end{array}$$

Therefore, this relation predicts that the quantum noise in the 410 nm InGaN laser is nearly 8.3 dB higher than that in the AlGaAs laser. This result fits the experimental finding by Matsuoka et al. [11] on 410 nm InGaN and 830 nm AlGaAs lasers with almost identical structure design and operation characteristics [11]. It is worth noting that the corresponding variation of the quantum noise level with the current ratio *I*/*I*_th_ reveals much smaller differences between both lasers, which agrees also with the experimental results in [11].

**Figure 5:**
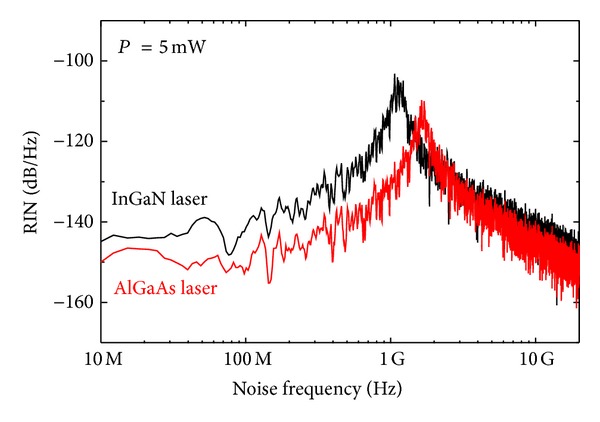
The spectra of RIN of InGaN and AlGaAs lasers when *P* = 5 mW.

Figure 5 compares the simulated spectrum of RIN of both lasers when *P* = 5 mW. As shown in the figure, the InGaN laser reveals RIN spectra higher than those of the AlGaAs laser. On the other hand, the position of the enhanced resonance peak of the InGaN laser occurs at a relaxation frequency lower than that of the AlGaAs laser.

### 4.3. Influence of Gain Saturation on RIN

**Figure 6:**
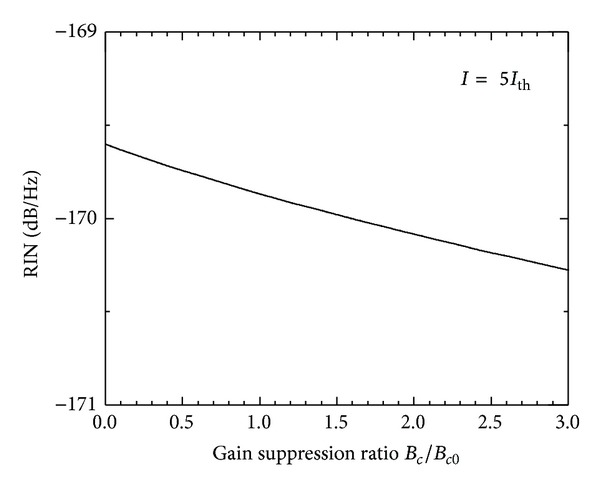
Influence of gain suppression factor on the LF-RIN level when *I* = 5*I*_th_.

The gain suppression is a critical property of the lasing medium and controls most of the dynamics and operation characteristics of semiconductor lasers. Influence of the nonlinear gain suppression *G*_*NL*_ on the quantum noise of the InGaN laser is illustrated in Figure 6 when *I* = 5*I*_th_. The gain suppression is varied by varying *B*_*c*_ relative to the set value in Table 1, *B*_*c*0_. The figure shows that the increase in *G*_*NL*_ results in a decrease in the LF-RIN level within 1 dB/Hz. The variation *G*_*NL*_ was found not to affect the position of the resonance peak of the RIN spectrum (i.e., the relaxation frequency). This suppression in the amplitude of intensity fluctuations agrees with the prediction by Abdulrhmann et al. [27] in InGaAsP lasers.

The spectral gain suppression is a nonlinear effect. In its most exact description, the optical gain is expressed as an infinite perturbation expansion of the field intensity [24]. Yamada and Suematsu [25] showed that within the normal operation of semiconductor lasers the gain suppression has partial homogeneous spectral broadening. Therefore, the gain of semiconductor lasers is usually described by the third-order perturbation form in (2) [25]. In semiconductor lasers with high power emission, the gain suppression becomes inhomogeneously broadened [25] and the infinite gain expansion in [23] is reduced to the following form developed by Agrawal [28]:

(9) $$\begin{array}{c} G=\frac{G_{L}}{\sqrt{1+\varepsilon S}}\;\text{where}\;\varepsilon =B_{c}\frac{V}{a\xi }. \end{array}$$

**Figure 7:**
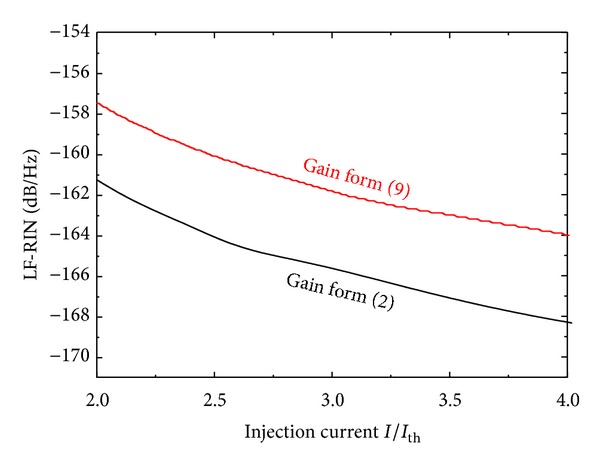
Dependence of LF-RIN on the injection current *I*/*I*_th_ of InGaN lasers under partial homogeneous gain broadening (form (2)) and inhomogeneous gain broadening (form (9)).

Here, we examine the validity of this gain suppression to model the noise properties of the present InGaN laser. Replacing form (2) of the partial homogeneous gain suppression with form (9) of the inhomogeneous gain suppression was found not to change the *L*-*I* characteristics. In Figure 7, we compare the obtained noise properties of the InGaN laser by those obtained when applying the form in (9). As shown in the figure, the inhomogeneous gain broadening little overestimates the RIN level in the regime of high injection currents; the difference is just within 4 dB.

## 5. Conclusions

We modeled and simulated the noise properties of the blue-violet InGaN laser diodes. We compared the RIN results with those of the AlGaAs lasers since both lasers are the most representative light sources in the high-density disc systems. Also, we examined the influence of the gain suppression on RIN. The results showed that the RIN spectra are flat in the low-frequency regime. RIN is suppressed remarkably with the increase in the injection current up to *I* ~ 1.6*I*_th_, beyond which the decrease in LF-RIN becomes within 0.5 dB/Hz. This increase in the injection level is associated also with an increase in the relaxation oscillation peak towards higher frequencies. The simulated noise results are in good agreement with the experimental data reported in literature. The RIN level in the InGaN laser is nearly 9 dB higher than that of the AlGaAs laser. A big contributor to this higher RIN is the inverse proportionality of RIN on the third power of the emission wavelength. The increase in the gain suppression was found to decrease the quantum noise within 1 dB/Hz. Describing the gain suppression by the form of inhomogeneous gain broadening was found to induce 4 dB overestimation of the RIN level.

## Figures and Tables

**Table 1:** Definition and numerical values of the InGaN laser parameters.

Symbol	Definition	Value
*λ*	Wavelength	410 nm
*n*_*D*_	Refractive index of active layer	2.6
*L*_*D*_	Active layer length	300 *μ*m
*a*	Differential gain coefficient	1.85 × 10^−12^ m^2^
*B*_*c*_	Nonlinear gain coefficient	2.7 × 10^−5^ s^−1^
*N*_*g*_	Carrier number at transparency	2.52 × 10^8^
*N*_*s*_	Carrier number characterizing *G*_NL_	2.01 × 10^8^
*α*	Linewidth enhancement factor	2
*τ*_*s*_	Spontaneous emission lifetime	2 ns
*R*_*f*_	Front facet reflectivity	0.4
*R*_*b*_	Back facet reflectivity	0.7
*G*_th_	Threshold gain	2.2 × 10^11^ s^−1^

## References

[1] Piprek J (2007). *Nitride Semiconductor Devices*.

[2] Nakamura S, Pearton S, Fasol G (2000). *The Blue Laser Diode*.

[3] Nakamura S, Senoh M, Nagahama S (1996). Optical gain and carrier lifetime of InGaN multi-quantum well structure laser diodes. *Applied Physics Letters*.

[4] Takeuchi T, Takeuchi H, Sota S, Sakai H, Amano H, Akasaki I (1997). Optical properties of strained AlGaN and GaInN on GaN. *Japanese Journal of Applied Physics*.

[5] Kobayashi T, Nakamura F, Naganuma K (1998). Room-temperature continuous-wave operation of GaInN/GaN multiquantum well laser diode. *Electronics Letters*.

[6] Kuramata A, Kubota S, Soejima R, Domen K, Horino K, Tanahashi T (1998). Room-temperature continuous wave operation of InGaN laser diodes with vertical conducting structure on SiC substrate. *Japanese Journal of Applied Physics*.

[7] Kuramoto M, Sasaoka C, Hisanaga Y (1999). Room-temperature continuous-wave operation of InGaN multi-quantum-well laser diodes grown on an n-GaN substrate with a backside n-contact. *Japanese Journal of Applied Physics*.

[8] Kneissl M, Bour DP, van de Walle CG (2006). Room-temperature continuous wave operation of InGaN. *Moldavian Journal of the Physical Sciences*.

[9] Ahmed M, Yamada M, Saito M (2001). Numerical modeling of intensity and phase noise in semiconductor lasers. *IEEE Journal of Quantum Electronics*.

[10] Yamada M (1994). Variation of intensity noise and frequency noise with the spontaneous emission factor in semiconductor lasers. *IEEE Journal of Quantum Electronics*.

[11] Matsuoka K, Saeki K, Teraoka E, Yamada M, Kuwamura Y (2006). Quantum noise and feed-back noise in blue-violet InGaN semiconductor lasers. *IEICE Transactions on Electronics*.

[12] Ahmed M (2003). Numerical characterization of intensity and frequency fluctuations associated with mode hopping and single-mode jittering in semiconductor lasers. *Physica D*.

[13] Ahmed M (2008). Spectral lineshape and noise of semiconductor lasers under analog intensity modulation. *Journal of Physics D: Applied Physics*.

[14] Abdulrhmann S, Ahmed M, Yamada M A new model of analysis of semiconductor laser dynamics under strong optical feedback in fiber communication systems.

[15] Smith AW, Armstrong JA (1966). Intensity noise in multimode GaAs laser emission. *IBM Journal of Research and Development*.

[16] Henry CH (1986). Phase noise in semiconductor lasers. *Journal of Lightwave Technology*.

[17] Agrawal GP, Olsson NA, Dutta NK (1984). Effect of fiber-far-end reflections on intensity and phase noise in InGaAsP semiconductor lasers. *Applied Physics Letters*.

[18] Agrawal GP Noise in semiconductor lasers and its impact on optical communication systems.

[19] Agrawal GP (1991). Effect of nonlinear gain on intensity noise in single-mode semiconductor lasers. *Electronics Letters*.

[20] Yamada M, Ishimori W, Sakaguchi H, Ahmed M (2003). Time-dependent measurement of the mode competition phenomena among longitudinal modes in long-wavelength Lasers. *IEEE Journal of Quantum Electronics*.

[21] Ahmed M (2004). Numerical approach to field fluctuations and spectral lineshape in InGaAsP laser diodes. *International Journal of Numerical Modelling: Electronic Networks, Devices and Fields*.

[22] Masoller C, Cabeza C, Schifino AS (1995). Effect of the nonlinear gain in the visibility of a semiconductor laser with incoherent feedback in the coherence collapsed regime. *IEEE Journal of Quantum Electronics*.

[23] Abdullah R (2014). The influence of gain suppression on dynamic characteristics of violet InGaN laser diodes. *Optik*.

[24] Ahmed M, Yamada M (1998). An infinite order perturbation approach to gain calculation in injection semiconductor lasers. *Journal of Applied Physics*.

[25] Yamada M, Suematsu Y (1981). Analysis of gain suppression in undoped injection lasers. *Journal of Applied Physics*.

[26] Ahmed M (2003). Numerical characterization of intensity and frequency fluctuations associated with mode hopping and single-mode jittering in semiconductor lasers. *Physica D: Nonlinear Phenomena*.

[27] Abdulrhmann S, Ahmed M, Yamada M (2002). Influence of nonlinear gain and nonradiative recombination on the quantum noise in InGaAsP semiconductor lasers. *Optical Review*.

[28] Agrawal GP (1986). Effect of gain nonlinearities on period doubling and chaos in directly modulated semiconductor lasers. *Applied Physics Letters*.

